# Inhibition of PLK3 Attenuates Tubular Epithelial Cell Apoptosis after Renal Ischemia–Reperfusion Injury by Blocking the ATM/P53-Mediated DNA Damage Response

**DOI:** 10.1155/2022/4201287

**Published:** 2022-06-24

**Authors:** Weiming Deng, Xiangling Wei, Zhenwei Xie, Rui Zhang, Zhanwen Dong, Jinhua Zhang, You Luo, Qingdi Cheng, Ruojiao Wang, Heng Li, Ning Na

**Affiliations:** ^1^Department of Kidney Transplantation, The Third Affiliated Hospital of Sun Yat-sen University, Guangzhou, Guangdong 510630, China; ^2^The First Affiliated Hospital, Department of Urology, Hengyang Medical School, University of South China, Hengyang, Hunan 421001, China

## Abstract

**Objective:**

Renal ischemia–reperfusion (I/R) injury is a major cause of acute kidney injury (AKI) in transplanted kidneys. This study was aimed at exploring the role of PLK3 (polo-like kinase 3) in renal I/R injury, focusing on its relationship with oxidative stress-induced DNA damage and renal tubular epithelial cell (TEC) apoptosis.

**Methods:**

TRAP-seq data from the development dataset GSE52004 and the validation dataset GSE121191 were analyzed using GEO2R. PLK3 overexpression plasmids and targeted silencing siRNAs were used in a model of hypoxia/reoxygenation (H/R) injury, and rAAV-9-PLK3-KD were administered to C57BL/6J mice exposed to I/R injury. The ATM-specific inhibitor KU-60019 was used to block the DNA damage response (DDR). Western blotting was performed to measure DDR- and apoptosis-associated protein expression. Cell viability was measured by CCK-8 reagent, and apoptosis was examined by flow cytometry and TUNEL assay. Furthermore, the fluorescent probes H_2_DCFH-DA and DHE were used to measure ROS production in vitro. The MDA level and SOD activity were measured to assess oxidative stress in vivo. KIM-1 staining and Scr and BUN were used to evaluate kidney injury.

**Results:**

The mRNA and protein levels of PLK3 were markedly increased in the H/R injury and I/R injury models. GO terms showed that PLK3 was mainly involved in oxidative stress and DNA damage after renal I/R injury. Overexpression of PLK3 decreased cell viability and increased apoptosis. In contrast, targeted silencing of PLK3 expression decreased the Bax/Bcl-2 ratio by decreasing P53 phosphorylation, thereby reducing TEC apoptosis. Furthermore, KU-60019 reduced PLK3 activation and DDR-induced apoptosis, while overexpression of PLK3 reversed the mitigating effect of KU-60019 on TEC apoptosis. Similarly, rAAV-9-PLK3 KD mice exhibited a lower rate of TEC apoptosis and milder renal damage after I/R injury.

**Conclusion:**

We demonstrate for the first time that PLK3 is involved in oxidative stress-induced DNA damage and TEC apoptosis in renal I/R injury. Inhibition of PLK3 attenuates TEC apoptosis after I/R injury by blocking the ATM/P53-mediated DDR. Therefore, PLK3 may serve as a potential therapeutic target for ischemic AKI.

## 1. Introduction

Acute kidney injury (AKI) is a common complication that is associated with high morbidity and mortality, and medications that are effective in treating AKI are lacking [1]. Ischemia–reperfusion (I/R) injury is an inevitable process associated with renal transplantation and the main cause of AKI after transplantation. When the kidney is exposed to hypoxia and ischemic injury, the proximal tubules are the most vulnerable part of the structure [2, 3].

The oxidative stress caused by the excessive production of reactive oxygen species (ROS) during reperfusion is generally considered to be the major cause of tubular epithelial cell (TEC) injury [4]. ROS attack DNA and cause DNA double-strand breaks (DSBs), which are one of the most critical DNA lesions with respect to TEC survival and death during oxidative stress [5, 6]. DSBs activate the protein kinase ataxia telangiectasia mutation (ATM), which initiates DNA damage response (DDR) signals to downstream target genes, affecting cell cycle arrest, DNA repair, and apoptosis [7]. P53 is an intracellular transcription factor that regulates the expression of genes involved in growth arrest or apoptosis in response to various stress conditions, including DNA damage and hypoxia [8]. After DNA damage, P53 protein stability is affected by posttranslational modification, including extensive phosphorylation. When DNA damage is severe and cannot be repaired, ATM stabilizes and activates P53 through phosphorylation at Ser15 and Ser20 to initiate the apoptosis cascade [9]. DNA damage occurs more frequently in proximal tubules than in distal tubules, and this trend may be related to subtle differences in sensitivity to damage and proliferation between these cell types [10]. Increasing evidence suggests that DNA damage and the related DDR play key roles in cisplatin-induced nephrotoxic AKI [11–13], but the current evidence on ischemic AKI remains poorly understood.

PLK3 is an evolutionarily conserved Ser/Thr protein kinase in the mammalian polo-like kinase (Plk) family that plays a vital role in the cell cycle and mitosis [14]. Unlike other PLKs, PLK3 acts as a tumor suppressor by blocking cell proliferation and inducing apoptosis [15–17]. PLK3 is often rapidly activated in response to various environmental stresses [18–20]. As an essential part of hypoxia regulation, PLK3 can directly phosphorylate HIF-1*α* and reduce its stability under hypoxic conditions [21]. Hypoxia/reoxygenation- (H/R-) induced PLK3 activation results in the direct phosphorylation of c-Jun, thereby promoting human corneal epithelial cell apoptosis [22]. When oxidative stress causes DNA damage, PLK3 can be phosphorylated in an ATM-dependent manner [23]. Therefore, the main function of PLK3 may be regulation of the stress response but not the cell cycle in mammals [24]. I/R injury-induced TEC apoptosis is a major factor in ischemic AKI in renal transplant patients. However, whether PLK3 is involved in the oxidative stress-induced DNA damage that leads to TEC apoptosis is still unclear.

In the current study, we provided compelling evidence that PLK3 promoted TEC apoptosis during ischemic AKI using a renal I/R injury model in vivo and a TEC H/R injury model in vitro. ROS generated by I/R injury caused DSBs, which activated PLK3 in an ATM-dependent manner, and then, PLK3 activated P53 to drive the DDR and apoptosis. Importantly, inhibiting PLK3 reduced apoptosis of TECs and attenuated renal injury in both the cellular and animal experiments. In conclusion, this study is the first to demonstrate that PLK3 plays a pivotal role in ischemic AKI and that inhibiting PLK3 may be a novel strategy for the prevention and treatment of renal I/R injury.

## 2. Materials and Methods

### 2.1. Bioinformatics Analysis

The gene expression profile GSE52004 was downloaded from the Gene Expression Omnibus (GEO) database (http://www.ncbi.nlm.nih.gov/geo/). The researchers developed a mouse line in which the translation profiles of specific cell types can be identified by utilizing cell-type specific CRE-driver lines and translating ribosomal affinity purification (TRAP) techniques. For this database, the researchers screened the cell-specific translational expression profiles of nephron (Six2), interstitial cell group (Foxd1), vascular endothelial cell (Cdh5), and macrophage/monocyte group (Lyz2) after renal I/R injury [25]. The TRAP-seq validation dataset was derived from GSE121191 [26]. Single-cell RNA-seq data from normal human kidney tissue were obtained from GSE131685 and analyzed with the Human Protein Atlas (https://www.proteinatlas.org/). We used the GEO2R online tool (http://www.ncbi.nlm.nih.gov/geo/geo2r/) to identify the differentially expressed genes (DEGs) and displayed these genes in the forms of a heatmap and column. The heatmap was generated by the imageGP (http://www.ehbio.com/ImageGP/index.php/Home/Index/index.html). To elucidate the function of PLK3 in renal I/R injury, the “clusterProfiler” R package was used to perform Gene Ontology (GO) functional enrichment analyses [27].

### 2.2. Cell Culture and the Induction of H/R In Vitro

The human proximal tubular cell line HK-2 was obtained from the American Type Culture Collection (Manassas, VA, USA) and validated by a short tandem repeat (STR) assay (IGE Biotech, Guangzhou, China). The cells were maintained in DMEM/F12 medium (Gibco, CA, USA) supplemented with 10% fetal bovine serum (Gibco, CA, USA), 100 IU/ml penicillin, and 100 *μ*g/ml streptomycin (Gibco, CA, USA) in a 37°C humidified incubator with an atmosphere of 95% air and 5% CO_2_. Before each experiment, HK-2 cells were seeded in six-well plates at a density of 1 − 5 × 10^5^ cells per well and cultured in complete DMEM/F12 medium until they reached approximately 70-80% confluence. To induce hypoxic injury, the medium was replaced with serum- and glucose-free DMEM/F12 medium (Procell, Wuhan, China), and the cells were exposed to hypoxic conditions (1% O_2_, 94% N_2_, and 5% CO_2_) in a humidified N_2_-flushed hypoxic chamber (Eppendorf, GALAXY-48R) for different times. After exposure to hypoxia, the cells were cultured in complete DMEM/F12 medium for different times under normoxic conditions (5% CO_2_ and 95% air) to allow reoxygenation. Control cells were maintained in complete medium in a normoxic cell incubator.

### 2.3. Animal Models of Renal I/R Injury

All the animal protocols were strictly conducted in accordance with institutional animal care policies and were approved by the Biomedical Ethics Committee of Sun Yat-sen University (Guangzhou, China). Male C57BL/6 mice (age 8-10 weeks and weight 22–25 g) were purchased from Guangdong Medical Experimental Animal Center. The mice were housed under pathogen-free conditions with a relative humidity of 50% and temperature of 25 ± 2°C and were maintained under conditions of 12 h of light and 12 h of darkness. To establish the model of renal I/R injury, the mice were anesthetized with pentobarbital sodium (60 mg/kg, i.p.) and placed on a heating pad to maintain a core body temperature of 37°C. A median abdominal incision was made, and the bilateral renal pedicles were blocked with a nontraumatic vascular clamp (FT222T, B. BRAUN, Germany) for 30 min. Then, the clamp was released, and reperfusion was visually confirmed. The sham operation group only underwent renal pedicle exposure without clamping.

### 2.4. Recombinant Adeno-Associated Virus Serotype-9- (rAAV9-) Mediated PLK3 Knockdown (KD) in Mice

rAAV-9 was developed by and obtained from GeneChem (Shanghai, China). All the mice were randomly divided into 4 groups (*n* = 6 per group): the sham group, saline+IRI group, rAAV9+IRI group, and rAAV9-PLK3-KD+IRI group. Before the operation, a 34 G microinjection needle was used to slowly inject 50 *μ*l of rAAV9-PLK3-KD plasmid at a concentration of 1 × 10^11^ vg/ml into the kidneys through the renal vein [28]. The animals were sacrificed 24 h after reperfusion, and blood and kidney tissue samples were collected for further analysis. The shRNA sequences were listed as follows: PLK3 shRNA sense (5′-3′): CTTTCTGGCCTCAAGTACT and control shRNA sense (5′-3′): CGCTGAGTACTTCGAAATGTC.

### 2.5. Analysis of Renal Function

Blood samples were collected from the submandibular vein, and then, the supernatant was collected after centrifugation. Creatinine and urea commercial kits (Nanjing Jiancheng Co., China) were used to calculate the levels of blood urea nitrogen (BUN) and creatinine according to the manufacturer's instructions.

### 2.6. Quantitative Real-Time PCR

Total RNA was extracted from HK-2 cells and mouse renal tissues with TRIzol reagent (Thermo Fisher Scientific, USA). The RNA yield was measured by NanoDrop 2000 (Thermo Scientific, USA). Subsequently, the HiScript III RT SuperMix+gDNA Wiper Kit (Vazyme, Nanjing, China) was used to reverse transcribe the RNA into cDNA. qRT–PCR analysis was performed using the ABI7500 system (Agilent Technologies, USA) with SYBR Green qPCR Master Mix (Vazyme, Nanjing, China). In all the PCR experiments, the expression levels of GAPDH (*Homo sapiens*) and *β*-actin (*Mus musculus*) were used as the internal references. Fold change in expression was quantified using the 2^−∆∆Ct^ method. The primers for the specific target genes (Supplementary table 1) were synthesized by IGE Biotech (Guangzhou, China).

### 2.7. Flow Cytometry

Cell apoptosis was examined by an Annexin-V-FITC/propidium iodide (PI) apoptosis detection kit (KeyGEN Biotech, Nanjing, China) after transfection and H/R treatment for the indicated times. HK-2 cells were collected and washed twice with phosphate-buffered saline (PBS). Then, the cells were stained with 5 *μ*l of Annexin V-fluorescein isothiocyanate (FITC) and 5 *μ*l of PI for 30 min in the dark at room temperature. A H_2_DCFH-DA fluorescent probe was used to measure the production of ROS with FITC parameter settings. Finally, the stained cells were scanned by an LSRFortessa™ flow cytometer (BD Biosciences, San Diego, CA, USA). The raw data were analyzed by FlowJo software (Tree Star Inc., Ashland, OR).

### 2.8. Cell Viability Assay

A Cell Counting Kit-8 (CCK-8) assay kit was used to assess cell viability. Transfected HK-2 cells were seeded in 96-well plates at a density of 5 × 10^3^ cells/well and subjected to H/R injury. The plates were incubated for 3 h at 37°C in the dark after 10 *μ*l of CCK-8 solution was added to each well. At the end of the experiments, a microplate reader was used to measure the absorbance at 450 nm. Cell viability was calculated as the percentage = [(*A* − *C*)/(*B* − *C*)] × 100% (*A*: OD value of treatment group, *B*: OD value of control group, and *C*: OD value of blank group).

### 2.9. Measurement of Oxidative Stress Index Levels

The level of malondialdehyde (MDA) and the activity of superoxide dismutase (SOD) in mouse kidney tissues were measured using commercial kits (Jiancheng, Nanjing, China). Intracellular ROS accumulation was examined with a DHE probe (Beyotime, Nanjing, China) according to the manufacturer's instructions. After transfection and H/R treatment, HK-2 cells were incubated with 5 *μ*mol/l DHE at 37°C for 30 min. Then, the cells were washed three times with serum-free cell culture medium, and red fluorescence was observed under a fluorescence microscope (Nikon, Tokyo, Japan). In addition, we examined the expression levels of PLK3 in a H_2_O_2_-induced oxidative stress model by stimulating HK-2 cells with different concentrations of H_2_O_2_ (50 *μ*M, 100 *μ*M, and 200 *μ*M) for 6 h.

### 2.10. Immunofluorescence Analysis

To perform the immunofluorescence assay, samples were fixed with 4% paraformaldehyde for 30 min at room temperature. After being washed with PBST, the samples were permeabilized with 0.3% Triton X-100 for 10 min and then blocked with 5% goat serum for 30 min. Subsequently, the samples were incubated with primary antibodies overnight at 4°C. The primary antibodies used in the present study were specific for the following proteins: PLK3 (DF4471, 1 : 250, Afftiny) and rH2AX (YP0218, 1 : 250, Immunoway). Then, the samples were washed 3 times with PBST and incubated with fluorescent secondary antibodies at room temperature for 1 h. DAPI was used to label the nucleus, and the cells were mounted with antifading mounting medium (Applygen, Beijing, China). Images were captured with a fluorescence microscope (Nikon, Tokyo, Japan).

### 2.11. TUNEL Assay

Apoptotic cells were analyzed by a TUNEL BrightGreen apoptosis detection kit (Vazyme, Nanjing, China) and a One Step TUNEL Apoptosis Assay Kit (Beyotime, Nanjing, China) according to the manufacturer's instructions. Samples were incubated with buffer containing FITC-12-dUTP and recombinant TdT enzyme for 1 h at 37°C. The number of apoptotic cells in randomly selected visual fields was counted and analyzed in a blinded manner under a fluorescence microscope.

### 2.12. Western Blotting

Mouse kidneys and HK-2 cells were lysed with cold lysis buffer (0.25 M NaCl, 50 mM Tris-HCl pH 7.4, 0.5% NP 40, 1 mM EDTA, 1 mM Na_3_VO_4_, 1 mM NaF, 1% cocktail, and 1 mM PMSF). BCA reagent was used to determine the total protein concentration. The separated proteins were then electrophoretically transferred to a polyvinylidene fluoride membrane. After blocking with 5% fat-free milk in PBST for 2 h at room temperature, the samples were incubated with primary antibodies overnight at 4°C. After being washed three times, the membranes were incubated with horseradish peroxidase-conjugated secondary antibodies at room temperature for 60 min. Finally, an ECL substrate (sc2048, Santa Cruz) was used to visualize the immunoreactive bands. The main antibodies used for Western blot analysis included PLK3 (4896S, 1 : 1000, Cell Signaling), PLK3 (DF4471, 1 : 1000, Afftiny), ATM (ab199726, 1 : 1000, Abcam), p-ATM (ab81292, 1 : 1000, Abcam), P53 (ab179477, 1 : 1000, Abcam), P53 (YT3528, 1 : 1000, Immunoway), p-P53 (9287S, 1 : 1000, Cell Signaling), rH2AX (YP0218,1 : 1000, Immunoway), Bcl-2 (BF9103, 1 : 1000, Affinity), and Bax (GB12-690, 1 : 1000, Servicebio); the relative expression of the targeted protein was normalized to that of *β*-actin (AC026, 1 : 5000, ABclonal).

### 2.13. Histopathological Evaluation and Immunohistochemistry

After establishing the I/R injury mouse model, 4% paraformaldehyde was infused into the hearts of the mice until the kidneys were completely exsanguinated. The kidneys were harvested, fixed with 4% paraformaldehyde at room temperature, embedded in paraffin, and stained with hematoxylin and eosin. Pathological sections were evaluated under a microscope by a nephropathologist in a blinded manner. Renal tubule damage, including renal tubule dilation, tubular epithelial damage, and cast formation, was scored by determining the percentage of necrotic tubules, and renal tubule damage was graded from 0 to 4 [29].

A commercial kit (ZSGB-BIO, Beijing) was used to perform immunohistochemical staining. Briefly, the sections were incubated with anti-KIM-1 (1 : 500) overnight at 4°C and then incubated with a secondary antibody (1 : 100) for 1 h at room temperature. For quantification, the ratio of optical density was calculated with ImageJ software (version 1.52) to assess the staining intensity.

### 2.14. Small Molecule Inhibitors, siRNAs, Plasmids, and Transfection

The specific ATM inhibitor KU-60019 was purchased from MCE Co., Ltd. (Shanghai, China). PLK3-siRNA was designed and synthesized by IGE Co., Ltd. (Guangzhou, China). The siRNA sequences are shown in Supplementary Table 1. The PLK3 overexpression plasmid was constructed with a pcDNA3.1 vector with a 3× Flag tag. Before transfection, HK-2 cells were seeded in 6-well plates and incubated at 37°C in 5% CO_2_ until they reached 60%-70% confluence. The siRNAs and plasmids were transfected by using Lipofectamine 2000 transfection reagent in Opti-MEM. The siRNA-NC and pcDNA3.1 vector served as the negative controls. After 6 h, the Opti-MEM was replaced with DMEM/F-12 medium supplemented with 10% FBS, and the cells were further cultured. Then, qRT–PCR and Western blot were used to evaluate gene knockdown and overexpression efficiency.

### 2.15. Statistical Analysis

R software version 4.0.4 was used for bioinformatics analysis. All statistical analyses were performed using GraphPad Prism 8.0 (GraphPad, La Jolla, CA, U.S.A.). Data are presented as the mean ± SD. Two-tailed unpaired *t*-test was used for comparisons between two groups, and one-way analysis of variance (ANOVA) was used for multigroup comparisons. A value of *P* < 0.05 was considered statistically significant.

## 3. Results

### 3.1. Identification of DEGs in Wild-Type (WT) Mice and Screened PLK3 in Four Distinct Cellular Subgroups

**Figure 1:**
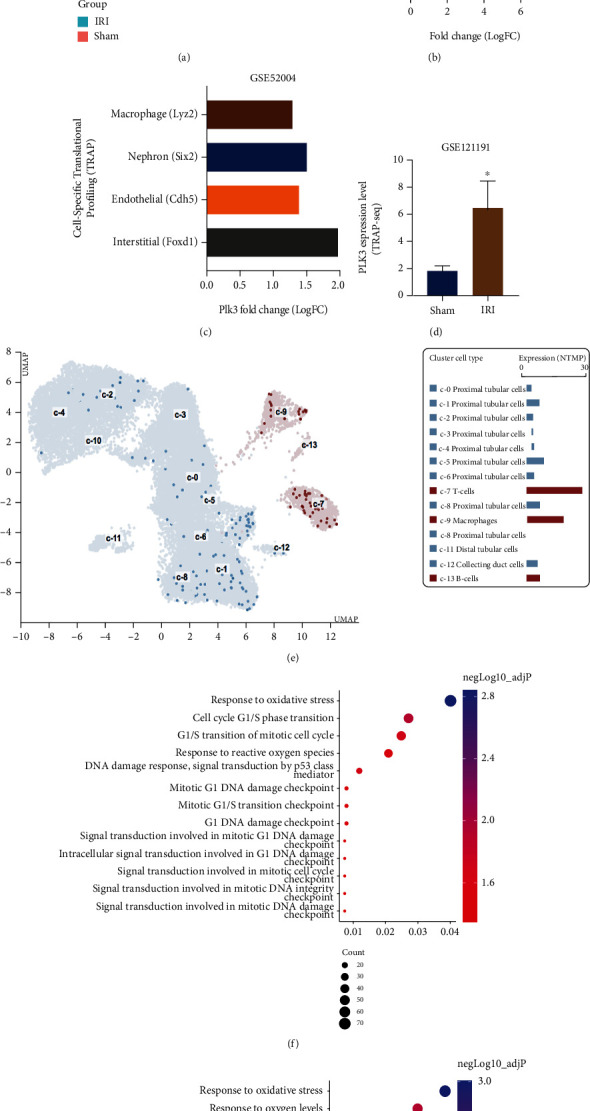
Bioinformatics analysis of PLK3 expression in renal TECs. (a) The heatmap shows the expression levels of the upregulated and downregulated DEGs (GSE52004). (b) Top 5 upregulated DEGs in TRAP-seq analysis of WT mice. (c) The expression level of PLK3 in four different cell subgroups 24 h after renal I/R injury. (d) The expression level of PLK3 in the TRAP-seq validation dataset (GSE121191) after renal I/R injury. (e) The expression level of PLK3 in single-cell RNA-seq data of normal kidney TECs derived from the Human Protein Atlas (GSE131685). (f) Bubble plots showing the enriched GO terms for PLK3-related biological processes in IRI vs. sham (TRAP-WT) DEGs. (g) Bubble plots showing the enriched GO terms for PLK3-related biological processes in IRI vs. sham (TRAP-Nephron) DEGs. The data are presented as the mean ± SD. *n* = 3. ^∗^*P* < 0.05.

A total of 821 (379 upregulated and 442 downregulated) DEGs were identified between the WT mice in the I/R injury (24 h) and sham groups from GSE52004 by GEO2R (|logFC| > 1.5, adj.*P* < 0.05) (Figure 1(a)). Interestingly, we found that PLK3 and the two most common markers of renal injury (Kim1 and Ngal) were among the top five upregulated DEGs (Figure 1(b)). PLK3 has not been previously described in studies of renal I/R injury, so we continued to compare the PLK3 translation profiles of four distinct cellular subgroups. PLK3 was highly expressed in all the cell groups, especially in interstitial cells (Foxd1) and TECs (Six2) (Figure 1(c)). The TRAP-seq validation dataset GSE121191 (adj.*P* < 0.05) also confirmed the significantly increased expression of PLK3 in renal TECs after 48 h of I/R injury (Figure 1(d)). Notably, the single-cell RNA-seq data from normal kidneys in the Human Protein Atlas indicated that the expression level of PLK3 in normal TECs was lower than that in immune cells (Figure 1(e)). GO enrichment analysis was used to verify the potential function of PLK3 in I/R injury. The TRAP-WT results suggested that PLK3 was significantly enriched in GO terms of biological processes, including oxidative stress, cell cycle, and DNA damage (Figure 1(f)). On the other hand, the TRAP-Nephron results suggested that PLK3 was mainly involved in the hypoxic response, oxygen level regulation, oxidative stress, and DNA damage response (Figure 1(g)). These biological processes are extremely important in ischemic AKI, suggesting that PLK3 may play an important role in renal I/R injury.

### 3.2. PLK3 Was Upregulated in Response to H/R Injury in TECs and I/R Injury in the Renal Cortex

**Figure 2:**
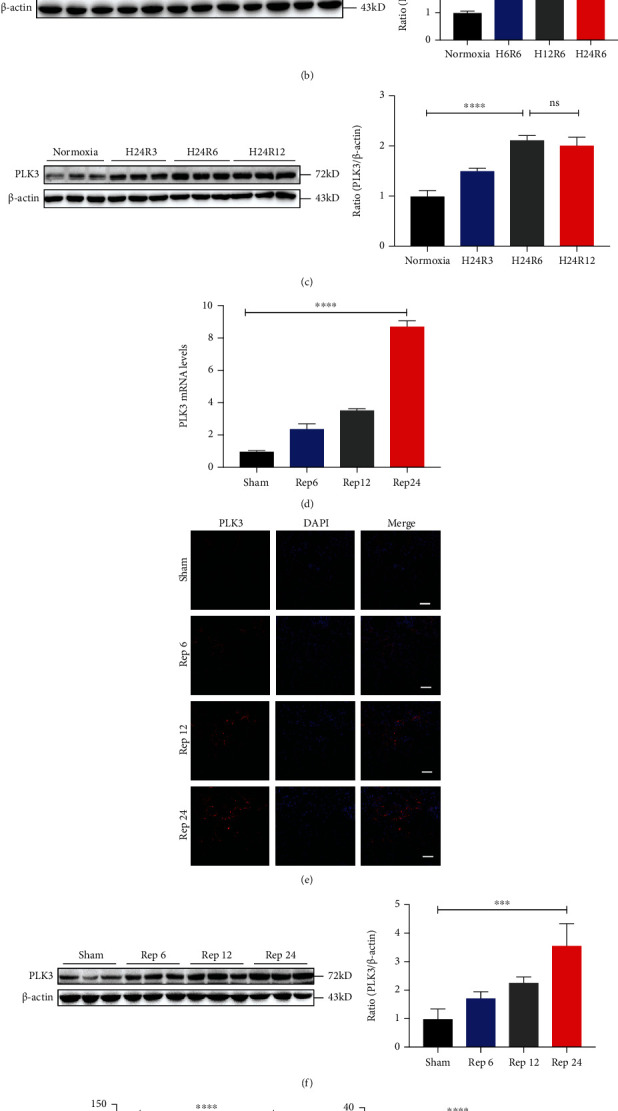
PLK3 expression was upregulated in TECs exposed to H/R injury and kidneys exposed to I/R injury. (a) PLK3 mRNA levels in HK-2 cells were measured by real-time RT–PCR after hypoxia and reoxygenation for different time periods. (b) PLK3 protein levels were measured by Western blot after different hypoxia time periods and the same reoxygenation time period. (c) PLK3 protein levels after the same hypoxia time period and different reoxygenation time periods. (d) PLK3 mRNA levels in the kidneys of C57BL/6J mice exposed to I/R were examined after 30 min of ischemia and different reperfusion times (6 h, 12 h, and 24 h). (e) PLK3 protein levels after different reperfusion times were observed by immunofluorescence (bar = 50 *μ*M; magnification, 400x). (f) PLK3 protein levels were measured after different reperfusion times. (g-h) Creatinine and BUN levels after different reperfusion times. The data are presented as the mean ± SD. *n* ≥ 3. ^∗^*P* < 0.05, ^∗∗^*P* < 0.01, ^∗∗∗^*P* < 0.001, and ^∗∗∗∗^*P* < 0.0001. BUN: blood urea nitrogen; H24R3: hypoxia for 24 h and reperfusion for 3 h; Rep24: ischemia for 30 min and reperfusion for 24 h.

We first examined the mRNA expression levels of PLK3 in HK-2 cells exposed to different hypoxic conditions and different reoxygenation times in the H/R model. The results showed that PLK3 mRNA expression was significantly higher in the H/R group than in the normoxia group, and its expression gradually increased with increasing hypoxia duration (Figure 2(a)). Similarly, PLK3 protein expression was also increased after H/R stimulation, and the highest protein level was observed after 24 h of hypoxia and 6 h of reoxygenation (Figure 2(b)). Since oxidative stress occurs mainly during the reoxygenation stage, we examined the changes in PLK3 expression in response to different reoxygenation times after 24 h of hypoxia, and the results demonstrated that PLK3 expression was higher at 6 h than at 3 h, and there was no additional increase after 6 h (Figure 2(c)). Therefore, 24 h of hypoxia and 6 h of reoxygenation were chosen as the time points for subsequent in vitro experiments.

In parallel, we examined the expression of PLK3 in the renal cortex by establishing a mouse model of I/R injury. Consistently, the mRNA and protein levels of PLK3 increased with increasing reperfusion time (Figures 2(d)–2(f)). Moreover, the kidney injury pathology score and enzymatic analyses results (creatinine and BUN levels) suggested that the kidney was most severely damaged at 24 h after the restoration of perfusion (Figures 2(g) and 2(h) and Supplementary Fig. 1A). In addition, the immunofluorescence results revealed a significant increase in PLK3-positive staining in renal tubules with increasing reperfusion time (Figure 2(e)). These results indicated that PLK3 was involved in the progression of renal I/R injury.

### 3.3. PLK3 Inhibition Enhanced Cell Viability and Reduced TEC Apoptosis in H/R Injury

**Figure 3:**
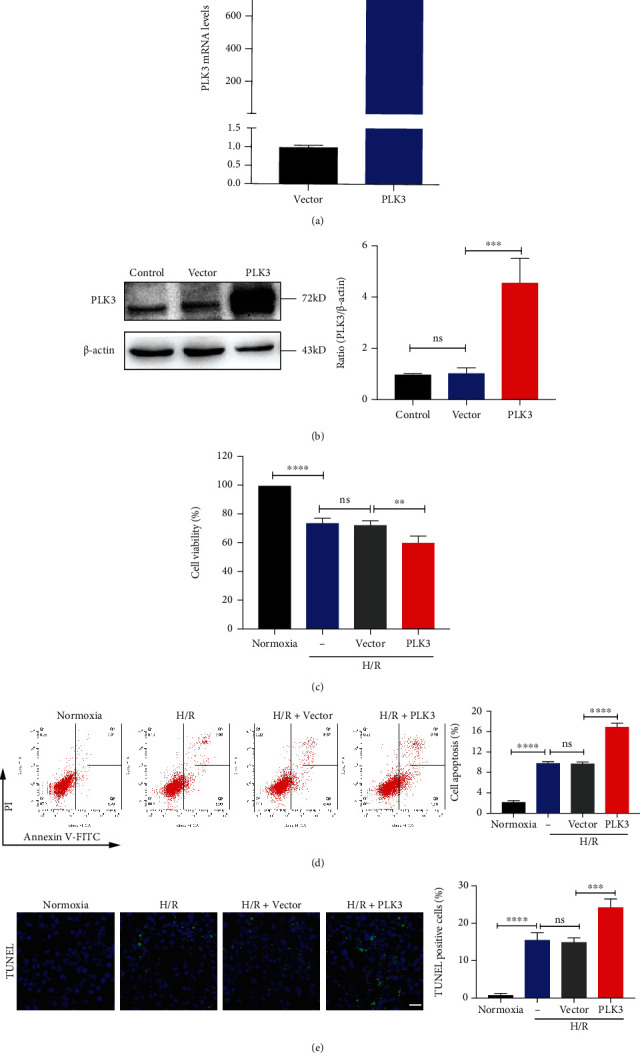
PLK3 overexpression promotes the TEC apoptosis caused by H/R injury. (a and b) Quantitative analysis and representative Western blot images of PLK3 mRNA and protein expression after transfection with the pcDNA3.1-flag-PLK3 plasmid. (c) CCK-8 reagent was used to evaluate the viability of HK-2 cells transfected with plasmids after H/R injury. (d) Representative graphs of the flow cytometry results showing apoptotic populations and quantitative analysis of apoptosis rates. (e) TUNEL staining to measure DNA fragmentation during apoptosis (bar = 50 *μ*M; magnification, 400x). The data are presented as the mean ± SD. *n* = 3. ^∗^*P* < 0.05, ^∗∗^*P* < 0.01, ^∗∗∗^*P* < 0.001, and ^∗∗∗∗^*P* < 0.0001.

**Figure 4:**
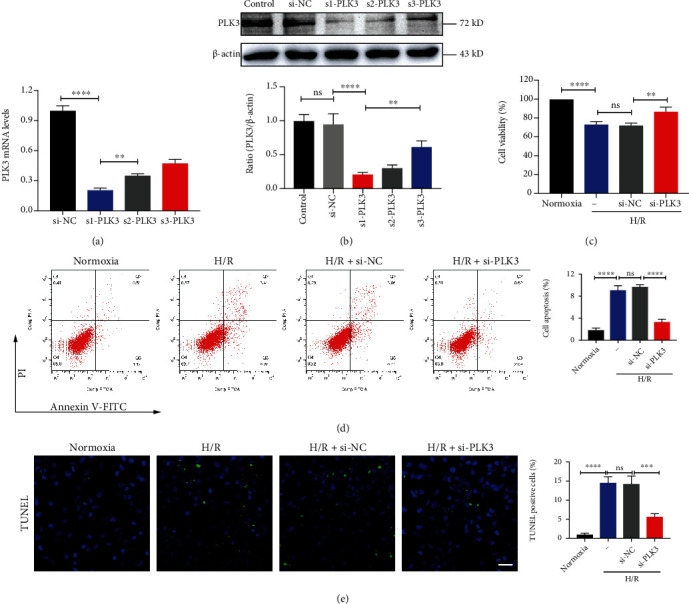
Targeted silencing of PLK3 expression reduces H/R-induced apoptosis in TECs. (a) qRT–PCR analysis of PLK3 mRNA expression after transfection with 100 nM PLK3-siRNA or scrambled oligonucleotide (si-NC). (b) Immunoblotting analysis of PLK3 silencing efficiency. (c) The viability of HK-2 cells was measured by CCK-8 assays. (d) Flow cytometry was performed to examine HK-2 cell apoptosis. (e) Representative images showing TUNEL-positive HK-2 cells after PLK3 silencing (bar = 50 *μ*M; magnification, 400x). The data are presented as the mean ± SD. *n* = 3. ^∗^*P* < 0.05, ^∗∗^*P* < 0.01, ^∗∗∗^*P* < 0.001, and ^∗∗∗∗^*P* < 0.0001.

We successfully constructed PLK3 overexpression plasmids and targeted siRNA sequences (Figures 3(a) and 3(b) and Figures 4(a) and 4(b)). First, a CCK-8 assay was used to measure cell viability. Compared with normoxic conditions, H/R injury significantly reduced the number of viable cells, while PLK3 overexpression further reduced cell viability (Figure 3(c)). However, silencing PLK3 expression enhanced cell viability under H/R conditions (Figure 4(c)). To investigate the effect of PLK3 on H/R-induced apoptosis of TECs, the incidence of apoptosis was assessed by flow cytometry and TUNEL assays. Flow cytometry revealed that PLK3 overexpression increased TEC apoptosis (Figure 3(d)). Notably, PLK3-targeted siRNA reduced the percentage of apoptotic cells observed after H/R injury (Figure 4(d)). TUNEL assays showed that the PLK3 plasmid increased the number of TUNEL-positive cells compared to the number of TUNEL-positive cells in the group exposed to H/R alone (Figure 3(e)), while inhibiting PLK3 expression decreased the number of apoptotic TECs (Figure 4(e)).

### 3.4. I/R Injury- and H/R Injury-Induced Exacerbation of Oxidative Stress and DNA Damage in TECs

**Figure 5:**
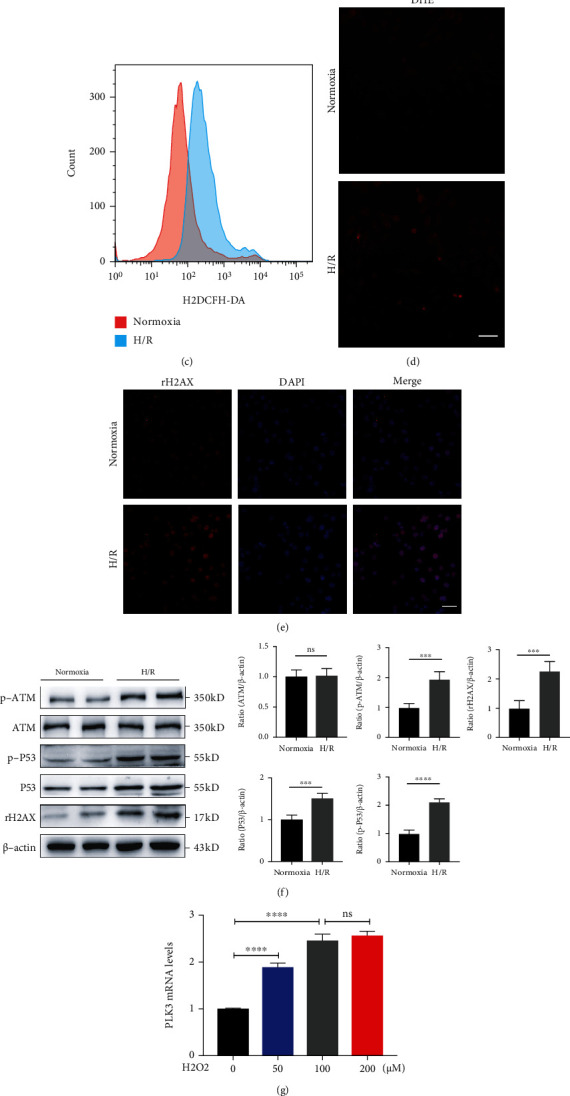
Oxidative stress and the DDR were induced during H/R injury in TECs and I/R injury in mouse kidneys. HK-2 cells were subjected to normoxia or hypoxia for 24 h/reoxygenation for 6 h. C57BL/6J male mice were subjected to sham operation or 30 min of bilateral renal ischemia, followed by 24 h of reperfusion. (a and b) MDA levels and SOD activity in renal cortex homogenates were analyzed. (c and d) Flow cytometric analysis of H_2_DCFH-DA staining and cytofluorometric analysis of DHE staining were performed to measure the levels of cellular ROS production after H/R treatment (bar = 50 *μ*M; magnification, 400x). (e) Representative images of immunofluorescence staining of *γ*H2AX in TECs (bar = 50 *μ*M; magnification, 400x). (f) The expression of DNA damage-related proteins was measured by Western blot after H/R injury. (g) PLK3 mRNA levels in the H_2_O_2_-induced oxidative stress model. (h) PLK3 protein levels after H_2_O_2_ stimulation. The data are presented as the mean ± SD. *n* = 3. ^∗^*P* < 0.05, ^∗∗^*P* < 0.01, ^∗∗∗^*P* < 0.001, and ^∗∗∗∗^*P* < 0.0001. DHE: dihydroethidium; MDA: malondialdehyde; SOD: superoxide dismutase.

We investigated the degree of oxidative stress in the I/R injury and H/R injury models. Compared with those in the sham-operated group, the MDA levels were increased and SOD activity was decreased in the renal cortex in the I/R injury group (Figures 5(a) and 5(b)). The results also showed that oxidative stress damage worsened with prolonged reperfusion time. Additionally, H_2_DCFH-DA and DHE staining in the H/R model further confirmed this result (Figures 5(c) and 5(d)). These findings suggested that I/R or H/R exacerbated oxidative stress in TECs. In mammalian cells, rH2AX is a marker of DSBs in response to DNA damage [30]. Immunofluorescence analysis suggested a marked increase in *γ*H2AX-positive TECs after H/R injury compared with that after exposure to normoxia (Figure 5(e)), and the Western blot results also confirmed significant upregulation of *γ*H2AX protein levels. As shown in Figure 5(f), the ATM phosphorylation and P53 phosphorylation levels were dramatically elevated by H/R injury, further suggesting that the DDR could be induced after H/R injury. In addition, in the H_2_O_2_-induced oxidative stress model, we stimulated HK-2 cells with different concentrations of H_2_O_2_, and the results showed that the expression of PLK3 was significantly increased (Figures 5(g) and 5(h)). In summary, these findings suggest that H/R injury can induce oxidative stress-mediated DNA damage, which may be an essential factor for TEC apoptosis.

### 3.5. Inhibiting PLK3 Attenuated TEC Apoptosis by Regulating P53-Mediated Apoptosis Pathways in Response to H/R Injury

**Figure 6:**
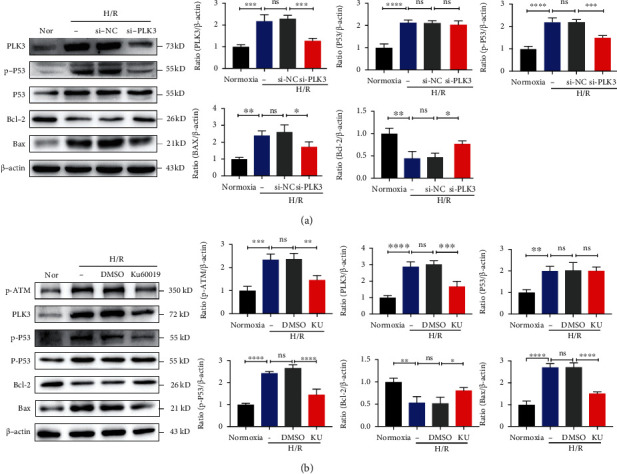
Inhibiting PLK3 attenuates TEC apoptosis via the ATM/P53 signaling pathway. (a) In the H/R injury model, the PLK3 protein levels and apoptosis-related protein levels were measured after targeted KD of PLK3 expression. (b) p-ATM protein levels, PLK3 protein levels, and apoptosis-related protein levels were measured by Western blot after the application of the specific ATM inhibitor KU-60019. The data are presented as the mean ± SD. *n* = 3. ^∗^*P* < 0.05, ^∗∗^*P* < 0.01, ^∗∗∗^*P* < 0.001, and ^∗∗∗∗^*P* < 0.0001. Nor: normoxia; Ku: KU-60019.

P53 can be activated to initiate apoptotic signaling after DNA damage. To investigate whether elevated PLK3 expression due to the DDR correlates with the phosphorylation and activation of P53, we examined the P53 phosphorylation levels in H/R-exposed TECs in which PLK3 expression was silenced. The results showed that low levels of PLK3 expression were accompanied by reduced P53 phosphorylation. Interestingly, knockdown of PLK3 expression did not alter the hypoxia-induced increase in total P53 levels. This result suggests that PLK3 acts as a protein kinase and is involved in the phosphorylation of P53 associated with the DDR. P53 activation may lead to apoptosis, and this effect is governed by a series of apoptotic genes, including Bcl-2 and Bax. Compared to cells cultured under normoxic conditions, cells exposed to H/R exhibited elevated expression of the proapoptotic gene Bax and decreased expression of the antiapoptotic gene Bcl-2. However, the expression of Bax in PLK3-KD TECs was significantly lower than that in si-NC-transfected cells, but Bcl-2 expression was elevated and the Bax/Bcl-2 ratio was reversed (Figure 6(a)). These results suggested that inhibiting PLK3 expression could attenuate downstream proapoptotic factors, leading to TEC apoptosis by reducing the phosphorylation of P53 during the DDR.

### 3.6. ATM Mediated the Induction of PLK3 in H/R Injury in TECs

ATM is one of the first sensors that is activated during the DDR. To determine whether ATM affects PLK3 expression, we used the specific ATM inhibitor KU-60019 and explored its inhibitory effect on PLK3 in HK-2 cells subjected to H/R for different times (Supplementary Fig. 2A). Western blot analysis showed that inhibiting p-ATM activation decreased the PLK3-induced P53 phosphorylation, thereby blocking the proapoptotic response downstream of P53 (Figure 6(b)). These results suggest that ATM, which is upstream of the DDR, can induce TEC apoptosis through the PLK3/P53 pathway in the presence of severe DNA damage.

### 3.7. The Alleviating Effects of ATM Inhibition on TEC Apoptosis Were Reversed by PLK3 Overexpression

**Figure 7:**
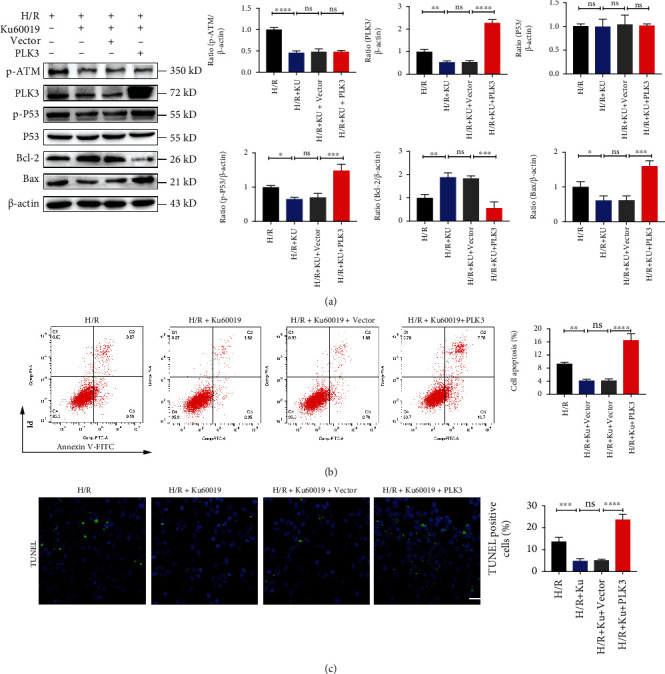
PLK3 overexpression reverses the effect of ATM inhibition in alleviating TEC apoptosis. (a) p-ATM protein levels, PLK3 protein levels, and apoptosis-related protein levels were measured by Western blot after the application of KU-60019 and PLK3 plasmid. (b) Flow cytometry was performed to examine the apoptosis of HK-2 cells after the administration of KU-60019 and PLK3 plasmids. (c) TUNEL assays showed the number of apoptotic HK-2 cells. The data are presented as the mean ± SD. *n* = 3. ^∗^*P* < 0.05, ^∗∗^*P* < 0.01, ^∗∗∗^*P* < 0.001, and ^∗∗∗∗^*P* < 0.0001.

We added KU-60019 to inhibit the DDR in HK-2 cells subjected to H/R injury and then transfected the cells with PLK3 overexpression plasmid. Western blot revealed no significant changes in the ATM phosphorylation levels, but the p-P53 levels were significantly increased, and the Bax/Bcl-2 ratio was elevated (Figure 7(a)). These results suggested that activation of PLK3, a downstream effector molecule of ATM, alone can also increase the phosphorylation of P53 to promote TEC apoptosis. In addition, we used flow cytometry and TUNEL assays to examine TEC apoptosis in vitro to verify these results (Figures 7(b) and 7(c)).

### 3.8. Knockdown of PLK3 Alleviated Renal Dysfunction and Tubular Damage in I/R-Induced AKI

**Figure 8:**
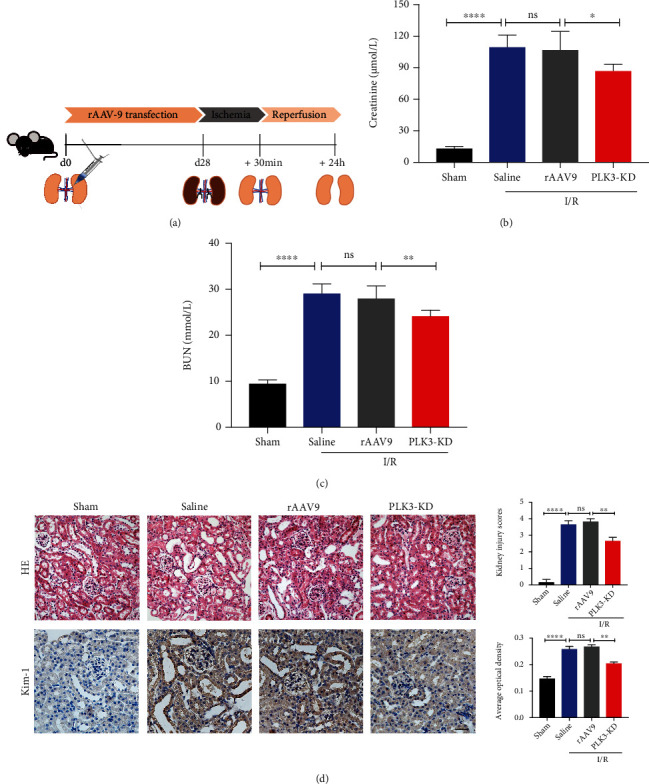
rAAV-9-mediated PLK3 KD attenuates I/R-induced AKI in mice. (a) Schematic diagram illustrating the animal experimental design. (b and c) Serum creatinine and BUN assays. (d) Sections of kidney tissue were stained with H&E, and tubular damage was quantified; KIM1 expression was examined by immunohistochemistry (bar = 50 *μ*M; magnification, 400x). The data are presented as the mean ± SD. *n* ≥ 3. ^∗^*P* < 0.05, ^∗∗^*P* < 0.01, and ^∗∗∗∗^*P* < 0.0001.

PLK3 expression was disrupted in C57BL/6J mice by renal vein injection of the rAAV9-PLK3-KD plasmid (Figure 8(a)). Real-time PCR and Western blot showed that the PLK3 mRNA and protein levels were significantly downregulated after 28 days (Supplementary Fig. 2B-C). Body weight measurements suggested that the injection of the rAAV-9 virus had no significant effect on body weight (Supplementary Fig. 2D). In mice suffering from I/R injury, the serum creatinine and BUN levels were significantly increased, while PLK3-KD decreased the serum creatinine and BUN levels after I/R injury (Figures 8(b) and 8(c)). Renal tubular damage is characterized by tubular dilatation, swelling, necrosis, and/or tubular congestion. Renal injury was evident in mice subjected to I/R injury, and PLK3-KD treatment alleviated this injury (Figure 8(d)). Immunohistochemical analysis of the expression of KIM-1, a marker of renal tubular injury, further confirmed that PLK3-KD attenuated I/R-induced AKI (Figure 8(d)). These results suggest that activating PLK3 in mice with I/R-induced AKI may be a key factor in susceptibility to AKI and that inhibiting PLK3 has a protective effect against ischemic AKI.

### 3.9. Inhibiting PLK3 Could Reduce TEC Apoptosis in I/R-Induced AKI

**Figure 9:**
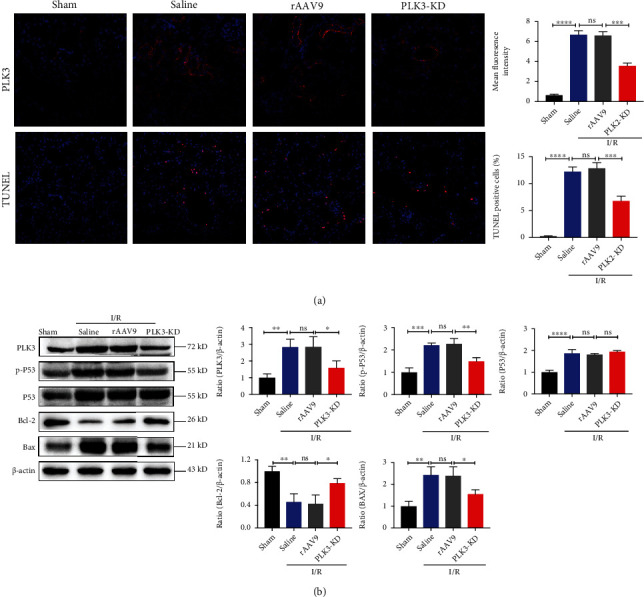
rAAV-9-mediated PLK3 KD alleviates I/R-induced TEC apoptosis in mice. (a) PLK3 expression was examined by immunofluorescence analysis; TUNEL analysis of apoptosis was performed (bar = 50 *μ*M; magnification, 400x). (b) Western blot analysis of PLK3 and apoptosis-related protein expression levels. The data are presented as the mean ± SD. *n* ≥ 3. ^∗^*P* < 0.05, ^∗∗^*P* < 0.01, ^∗∗∗^*P* < 0.001, and ^∗∗∗∗^*P* < 0.0001.

Immunofluorescence was performed to measure the expression of PLK3 in the renal cortical region, and the results suggested that PLK3-positive staining was diminished in PLK3-KD mice compared with rAAV9 mice. The TUNEL assay was used to examine genomic DNA fragmentation during apoptosis, and the results revealed significantly decreased positive staining in the renal cortex of PLK3-KD mice, indicating that blocking PLK3 expression reduced apoptosis and DNA fragmentation in TECs (Figure 9(a)). Moreover, we extracted the renal cortex, which contains a large number of nephrons, from mice with I/R-induced AKI for Western blot. The results indicated that I/R injury enhanced P53 phosphorylation and Bax expression and decreased Bcl-2 expression compared with the control, whereas PLK3-deficient mice showed significantly reduced P53 phosphorylation and Bax expression and enhanced Bcl-2 expression after I/R injury (Figure 9(b)). These results suggest that inhibiting PLK3 can reduce DNA damage and thus protect against TEC apoptosis during ischemic AKI.

## 4. Discussion

In a complex tissue such as the kidney, the high degree of cellular heterogeneity complicates the analysis of gene expression profiles derived from whole tissues. Comprehensive transcriptional profiles and gene expression profiles of specific cell types in complex tissues are essential for understanding the roles of genes in different cells [31]. The advantage of TRAP versus other gene expression profiling methodologies is that it allows in situ analysis of the mRNA profile of any genetically defined cell type, and the results match protein expression results more closely than the results of total RNA gene expression profiling [32]. Renal tubules are portion of the kidney that is most vulnerable to I/R injury, and renal tubules contribute most strongly to renal failure. To better characterize the molecular and cellular events related to AKI, we used the TRAP-seq results in the GEO database to examine PLK3 expression in the translation profile of nephrons (tubules) exposed to IRI for 24 h.

GO analysis showed that PLK3 was mainly involved in oxidative stress and DNA damage after renal I/R injury. DNA damage has been reported to be involved in I/R injury in a variety of vital organs, including the testicular, brain, liver, and heart [33–36]. During renal I/R injury, DNA fragmentation in the renal tubules after DNA damage occurs as early as 12 h after reperfusion and increases within 24 h [37]. In vitro, significant DNA damage occurs in rat proximal renal TECs after 5 min of hypoxia and 30 min of reoxygenation, and DSBs are exacerbated in a time-dependent manner [38]. AKI caused by sepsis, ischemia, or nephrotoxic drugs can lead to rapidly progressive renal dysfunction. Genomic DNA damage plays a critical role in the phenotypic changes in TECs and the subsequent loss of renal function. In nephrotoxic and ischemic AKI, TRIP13 deletion leads to increased DNA damage and activation of apoptotic signaling, resulting in TEC injury [39]. Endogenous salbutamol-*β* treatment exacerbates sepsis-related and nephrotoxic AKI by activating the ROS/DNA damage/P53 apoptotic pathway [11]. CCN2 exacerbates ischemic AKI by increasing the expression of *γ*H2AX and p-P53 through oxidative DNA damage [40].The degree of DNA damage is inversely correlated with the estimated glomerular filtration rate (eGFR) [41]. Therefore, activation of the DDR signaling pathway may represent a common mechanism that leads to AKI. Studies have confirmed that HMSCs and high-dose vitamin B12 protect renal function after I/R injury by reducing renal superoxide levels and lipid peroxidation, thereby attenuating the DDR and TEC apoptosis [42, 43]. After renal I/R injury, application of BA (boric acid) decreased the degree of DNA damage by preventing partially oxidative stress [44]. Our current study also confirmed that inhibition of PLK3, a phosphokinase directly involved in the DNA damage cascade response, protects TECs from renal I/R injury.

PLK3 has never been examined in the context of renal I/R injury. In the present study, we found significant upregulation of PLK3 expression in renal TECs in response to H/R injury and renal I/R injury. We observed renal injury and PLK3 expression changes in the I/R model with bilateral renal ischemia for 30 min and reperfusion for different times (6 h, 12 h, and 24 h). With the prolongation of reperfusion time, the expression of PLK3 showed the same trend as the indicators of renal function (creatinine and BUN), suggesting that the expression of PLK3 was related to the severity of renal injury. When cells are subjected to I/R injury, large amounts of ROS are produced, which disrupt the balance between oxidative and antioxidant systems and ultimately lead to oxidative stress [45]. ROS induced by I/R injury in vivo or H/R injury in vitro can lead to a series of severe damaging effects, such as lipid peroxidation, mitochondrial damage, and increased susceptibility to renal tubular injury [46]. Similarly, DNA damage due to H/R injury in vitro is mainly caused by oxidative stress associated with the reoxygenation-induced production of ROS [47]. To confirm the occurrence of oxidative stress and DNA damage in our in vitro model, we used a ROS fluorescent probe to measure ROS production and immunofluorescence analysis to observe rH_2_AX staining. The results showed significant oxidative stress and DSBs. To further confirm that PLK3 can be activated by oxidative stress, we measured PLK3 expression and observed significantly elevated PLK3 expression in H_2_O_2_-stimulated HK-2 cells.

ATM plays dual roles in DNA damage due to I/R. It plays a protective role during ischemic preconditioning but promotes cell death in lethal ischemic injury [48]. As one of the key transducers of the DDR, ATM undergoes rapid autophosphorylation at serine 1981 after DNA damage; then, ATM is recruited to the site of DNA damage to initiate repair, but in the context of severe DNA damage, it initiates apoptosis [49]. In this study, we confirmed that ATM was rapidly phosphorylated at serine 1981, which led to the activation of the downstream kinase PLK3 during H/R injury. We confirmed that PLK3 overexpression exacerbated TEC apoptosis after H/R injury, while targeted silencing of PLK3 expression reduced apoptosis. Specific ATM inhibitors attenuated apoptosis in the presence of multiple DNA damage-inducing factors [50, 51]. Compared with KU-55933, KU-60019 is an improved ATM kinase-specific inhibitor that has a more potent inhibitory effect [52]. KU-60019 reduced the protein levels of p-ATM and PLK3 and reduced the phosphorylation of P53, thereby regulating the downstream Bax/Bcl-2 ratio and attenuating apoptosis. In the rescue assay, PLK3 overexpression reversed the cytoprotective effect of KU-60019 on the DDR. Furthermore, we found that cell viability decreased after H/R injury, which may be related to the significant decrease in the nucleotide levels after hypoxia and the decrease in replication caused by DNA damage [53]. Our results suggest that silencing PLK3 expression during DDR injury can improve the viability of TECs.

**Figure 10:**
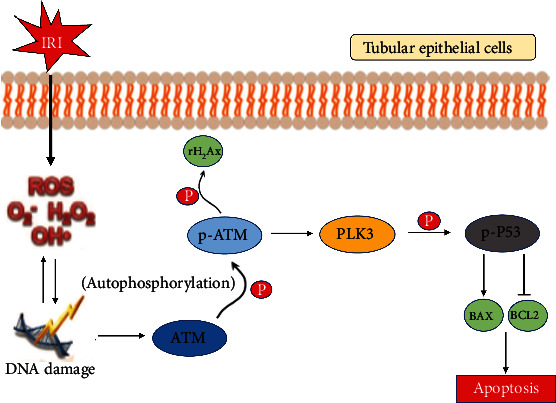
A schematic representation of the proposed mechanisms by which PLK3 regulates TEC apoptosis after DNA damage in response to oxidative stress. Renal I/R injury causes severe oxidative stress, and ROS attack double-stranded DNA, resulting in DSBs, which activate the ATM/PLK3/P53 signaling pathway in response to DNA damage, triggering the P53-dependent downstream apoptotic cascade, and thereby promoting TEC apoptosis.

Oxidative DNA damage, which can be induced by the ROS that are generated after renal I/R injury, directly induces TEC apoptosis [11, 54]. The TUNEL assay can detect DNA double-stranded and single-stranded breaks, which are markers of apoptosis after DNA damage [55]. Consistently, we found that H/R-induced apoptosis was accompanied by oxidative stress and the DDR. Accumulating evidence suggests that DNA damage and apoptosis are important steps in the pathogenesis of a variety of renal diseases [56, 57]. Consistent with previous studies [58], we also found that P53 protein expression in renal tubules was very low under baseline physiological conditions and increased significantly after ischemic injury. P53 is the master player in DDR-induced apoptosis, and the phosphorylation of P53, rather than the total expression of this protein, is the key to the induction of apoptosis [59]. DNA damage signaling induces P53 phosphorylation and stabilization and enhances its robust transcriptional activity to initiate the apoptosis cascade [60]. In the intrinsic mitochondrial apoptotic pathway, the activation of Bax by P53 transcription leads to permeabilization of the outer mitochondrial membrane and the release of apoptotic factors, which further activate caspases to induce apoptosis. Conversely, Bcl-2 antagonizes Bax to prevent mitochondrial permeabilization and inhibit apoptosis. In our study, we confirmed that H/R injury and I/R injury significantly increased the level of P53 phosphorylation. After P53 transcriptional activity is enhanced, expression of the downstream proapoptotic factor Bax is also significantly increased, and expression of the survival protein Bcl-2 is reduced; thus, the intrinsic apoptosis cascade is initiated in TECs. However, targeted silencing of PLK3 expression in vitro or knockdown of PLK3 expression with rAAV-9 plasmids in vivo could block P53-mediated apoptosis by reducing the phosphorylation of P53 to alleviate ischemic AKI and protect renal function. These results suggest that PLK3 functions as a protein kinase in the DDR and regulates DNA damage-induced apoptosis by phosphorylating its substrate P53 (Figure 10). This study provides insight into the causal relationship among oxidative stress, oxidative DNA damage, and TEC apoptosis in renal I/R injury.

## 5. Conclusions

In summary, this study is the first to show that PLK3 is activated during renal I/R injury and associated with oxidative stress and DNA damage. Oxidative stress caused by excessive ROS production can lead to DSBs, which can activate ATM, as well as downstream signaling responses to DNA damage. PLK3 activation by phosphorylated ATM increases the phosphorylation of P53, which enhances the P53-mediated proapoptotic cascade to promote TEC apoptosis. Overall, inhibiting PLK3 attenuates renal I/R injury-induced apoptosis by blocking the ATM/P53-mediated DDR, suggesting that PLK3 could be the target of effective therapeutic strategies to treat I/R injury during kidney transplantation.

## Data Availability

Data are available at NCBI GEO: GSE52004 and GSE121191.

## Supplementary Materials

Supplementary Materials Table 1: the primer sequence of PLK3 siRNA. Table 2: the primer sequence of qPCR. Supplementary data to this article can be found online. Figure 1: (A) renal tissues for H&E staining and quantification of tubular damage (bar = 50 *μ*M; magnification, 400x). (B and C) TUNEL assays showed the number of apoptotic after I/R injury (bar = 50 *μ*M; magnification, 400x). The data are presented as the mean ± SD. *n* ≥ 3. ^∗^*P* < 0.05, ^∗∗^*P* < 0.01, and ^∗∗∗∗^*P* < 0.0001. PC: positive control; NC: negative control. Supplementary data to this article can be found online. Figure 2: (A) p-ATM protein levels, PLK3 protein levels were measured by Western blotting after the application of KU-60019. (B and C) PLK3 expression after I/R injury at day 21 and day 28 of rAAV9-PLK3-KD plasmid injection. (D) PLK3 mRNA expression after rAAV9-PLK3-KD plasmids injection was detected of by qPCR. (E) Body weight measurement of mice injected with rAAV9 vector and rAAV9-PLK3-KD plasmids. The data are presented as the mean ± SD. *n* ≥ 3. ^∗^*P* < 0.05, ^∗∗∗^*P* < 0.001, and ^∗∗∗∗^*P* < 0.0001. 21d: 21 days; 28d: 28 days. Supplementary data to this article can be found online.
