# Supplementary material for: Inhibition of PLK3 Attenuates Tubular Epithelial Cell Apoptosis after Renal Ischemia–Reperfusion Injury by Blocking the ATM/P53-Mediated DNA Damage Response
Source: Oxid Med Cell Longev. 2022 Jun 24;2022:4201287. doi: 10.1155/2022/4201287 (PMC9249506; doi:10.1155/2022/4201287)
Supplement: Supplementary Materials — Table 1: the primer sequence of PLK3 siRNA. Table 2: the primer sequence of qPCR. Supplementary data to this article can be found online. Figure 1: (A) renal tissues for H&E staining and quantification of tubular damage (bar = 50 μM; magnification, 400x). (B and C) TUNEL assays showed the number of apoptotic after I/R injury (bar = 50 μM; magnification, 400x). The data are presented as the mean ± SD. n ≥ 3. ∗P < 0.05, ∗∗P < 0.01, and ∗∗∗∗P < 0.0001. PC: positive control; NC: negative control. Supplementary data to this article can be found online. Figure 2: (A) p-ATM protein levels, PLK3 protein levels were measured by Western blotting after the application of KU-60019. (B and C) PLK3 expression after I/R injury at day 21 and day 28 of rAAV9-PLK3-KD plasmid injection. (D) PLK3 mRNA expression after rAAV9-PLK3-KD plasmids injection was detected of by qPCR. (E) Body weight measurement of mice injected with rAAV9 vector and rAAV9-PLK3-KD plasmids. The data are presented as the mean ± SD. n ≥ 3. ∗P < 0.05, ∗∗∗P < 0.001, and ∗∗∗∗P < 0.0001. 21d: 21 days; 28d: 28 days. Supplementary data to this article can be found online. [file 4201287.f1.zip › Supplementary table1.doc]

**Table 1. The primer sequence of PLK3 siRNA.**

| **Target gene** | **Primer** | **Primer sequence (5’-3’)** |
| --- | --- | --- |
| Homo-PLK3-siRNA-1#  Homo-PLK3-siRNA-2#  Homo-PLK3-siRNA-3#    Homo-siRNA-NC | Forward  Reverse  Forward  Reverse  Forward  Reverse  Forward  Reverse | GCGAGAAGAUCCUAAAUGA  UCAUUUAGGAUCUUCUCGC  GCAGAAAGAAGAAGAGUAA  UUACUCUUCUUCUUUCUGC  GCAUCAAGCAGGUUCACUA  UAGUGAACCUGCUUGAUGC  UUCUCCGAACGUGUCACGU  ACGUGACACGUUCGGAGAA |

**Table 2. The primer sequence of qPCR.**

| **Target gene** | **Accession number** | **Primer** | **Primer sequence (5’-3’)** | **Product** |
| --- | --- | --- | --- | --- |
| *Mus*-PLK3  *Mus*-GAPDH  *Homo*-PLK3  *Homo*-GAPDH | NM_013807  NM_001289726  NM_004073  NM_001357943 | Forward  Reverse  Forward  Reverse  Forward  Reverse  Forward  Reverse | TGGGCATCCTGCGGTATTTTG  CCGTAGAAGTTCACCTGGACA  AGGTCGGTGTGAACGGATTTG  TGTAGACCATGTAGTTGAGGTCA  GCCTACGCTGTCAAAGTCAT  GCTCAATCTCATTTAGGATCTTCTC  GTCTCCTCTGACTTCAACAGCG  ACCACCCTGTTGCTGTAGCCAA | 178bp  123bp  79bp  131bp |
